# Preconditioning with hydrogen gas produces cardioprotective effects through autophagy activation in rat cardiomyocytes

**DOI:** 10.1186/s40360-025-01032-9

**Published:** 2025-11-26

**Authors:** Tokuhiro Yamada, Aya Kimura, Takashi Juri, Koichi Suehiro, Takashi Mori

**Affiliations:** Department of Anesthesiology, Osaka Metropolitan University Graduate School of Medicine, 1-5-7 Asahimachi, Abenoku, Osaka City, Osaka, 545-8586 Japan

**Keywords:** Hydrogen gas, Cardiac preconditioning, Mitochondrial permeability transition pore, Extracellular signal-regulated kinase, Autophagy, Microtubule-associated light chain 3-II

## Abstract

**Background:**

It is controversial whether hydrogen (H_2_) gas can activate cellular survival cascades and autophagy in the myocardium. This translational study aimed to investigate the hypothesis that preconditioning with inhaled H_2_ gas had a cardio-protective effect mediated through the activation of cellular signaling cascades and autophagy.

**Methods:**

Rat cardiomyocytes were isolated following 3% H_2_ gas inhalation for 60 min and were then incubated in a solution with or without 10 μM of 3-methyladenine (MA), an autophagy inhibitor. Intracellular Ca^2+^ concentration after 10-min perfusion of hydrogen peroxide, Ca^2+^ mobilization from the endoplasmic reticulum (ER), and opening of the mitochondrial permeability transition pore (MPTP) were estimated using fluorescence imaging. The expressions of extracellular signal-regulated kinase (ERK) and Akt and the levels of mitochondrial membrane potentials (ψm) were determined using flow cytometry. Furthermore, autophagosomes and microtubule-associated light chain (LC) 3-II were estimated using for autophagy activation flow cytometry.

**Results:**

H_2_ gas inhalation effectively inhibited the increase in the intracellular Ca^2+^ concentration and Ca^2+^ mobilization from the ER. The inhibitory effects of H_2_ gas were completely abolished by pretreatment with 3-MA. H_2_ gas inhalation also prolonged the time to MPTP opening, which was considerably shortened by the 3-MA pretreatment. The expression of signaling molecules and the levels of ψm decreased after 1.8 mM Ca^2+^ stimulation. Regardless of the stimulation, H_2_ gas inhalation preserved the ERK expression and ψm levels and increased autophagosomes and the LC3-II expression, but it did not affect the Akt expression. The 3-MA pretreatment completely abolished the cardio-protective effects of H_2_ gas as well as the autophagy activation.

**Conclusions:**

Preconditioning with H_2_ gas confers the cardio-protective effect in association with the activation of the mitogen-activated protein kinase/ERK pathway and autophagy in the myocardium. Thus, it may be feasible and effective as preventive intervention in patients undergoing cardiac surgery.

## Introduction

Ischemia/Reperfusion (I/R) injury is a major adverse event in patients undergoing cardiovascular surgery and is associated with massive production of reactive oxygen species (ROS) during reperfusion [1]. Most importantly, superoxide-derived hydroxyl radicals cause severe cellular damage because of their highly oxidizing properties. Hydrogen (H_2_) gas is a potential therapeutic agent against oxidative stress, because it selectively scavenges cytotoxic hydroxyl radicals but does not eliminate several ROS that possess physiological activities [2]. The feasibility and effectiveness of the inhalation approach could promote the clinical application of H_2_ gas in pathological states associated with massive ROS generation. Several clinical trials have mentioned that H_2_ gas inhalation can reduce I/R injury-induced myocardial damage in post-cardiac arrest syndrome or percutaneous coronary intervention [3, 4].

In addition to its scavenging properties against hydroxyl radicals, H_2_ gas can potentially activate several signaling pathways and mitigate I/R injury-induced myocardial damage [5]. Several signaling molecules, such as extracellular signal-regulated kinase (ERK) and Akt, are upstream factors that regulate the mitochondrial permeability transition pore (MPTP) to produce a preconditioning effect against subsequent cardiac damage [6, 7]. In addition to signaling pathways, autophagy has been recently shown to preserve mitochondrial homeostasis and confer cellular protection in cardiovascular diseases [8]. ERK and Akt are suggested to play major roles in regulating autophagy-related processes [9, 10]. Therefore, H_2_ gas inhalation may be a breakthrough treatment that induces the interaction of signaling pathways and autophagy to preserve cellular and mitochondrial homeostasis and confer the preconditioning effect in the myocardium.

Thus far, it is unclear whether H_2_ gas inhalation activates cellular cascades that mediate a preconditioning effect in the myocardium, although this intervention can potentially activate several signaling pathways. Similarly, it is controversial whether inhaled H_2_ gas activates autophagy-related processes. As very few researches have investigated the association between H_2_ gas and signaling cascades for modulating autophagy in the myocardium, this translational study aims to clarify whether preconditioning with inhaled H_2_ gas has a cardio-protective effect derived from the interaction between activated signaling pathways and autophagy. We hypothesized that H_2_ gas inhalation, together with the combined activation of cellular signaling cascades and autophagy, has a cardiac preconditioning effect against subsequent cardiac damage. Our findings could provide insight into the preconditioning effect of H_2_ gas against I/R injury-induced myocardial damage and the clinical significance of preventive interventions in perioperative medicine, especially in patients undergoing cardiac surgery.

## Methods

### Cell preparation

Six-week-old male Sprague-Dawley rats were purchased from an animal laboratory (CLEA Japan, Inc., Tokyo, Japan) and fed *ad libitum* under pathogen-free conditions until they attained a body weight of 300–350 g. In view of flammability, 3% concentration of H_2_ gas was prepared by mixing 4% H_2_ (Taiyo Nippon Sanso Co., Tokyo, Japan) and 100% oxygen (O_2_) in the ratio of 3:1. Prior to all experimental protocols, the rats inhaled 4 L·min^−1^ of 3% H_2_ or the mixture of nitrogen and O_2_ in the same ratio for 60 min in an experimental cage with an exhaust outlet, where H_2_ and O_2_ concentrations were continually monitored (GX-8000, Riken Keiki Co., Ltd., Tokyo, Japan).

After a 30-min stabilization, the hearts were harvested and isolated as previously described [11]. Briefly, rats were intraperitoneally administered with pentobarbital sodium (40 mg/kg, [Sigma-Aldrich, Inc., St. Louis, MO, USA]), xylazine hydrochloride (10 mg·kg^−1^, [Sigma-Aldrich, Inc.]), and heparin sodium (500 IU·kg^−1^, [Sigma-Aldrich, Inc.]). The isolated hearts were cannulated from the aorta and perfused with a normal Tyrode’s solution consisting of 140 mM NaCl, 5.4 mM KCl, 1.8 mM CaCl_2_, 0.33 mM NaH_2_PO_4_, 0.5 mM MgCl_2_, 5.0 mM HEPES, and 5.5 mM glucose at 37 °C. After perfusion with a Ca^2+^-free Tyrode’s solution containing 0.08% collagenase (FUJIFILM Wako Pure Chemical Co., Osaka, Japan), the left ventricle was minced and centrifuged at 400 ×g for 3 min. The pellet was repeatedly suffused and stabilized for 30 min at 23 °C (room temperature).

### Experimental allocation

**Fig. 1:**
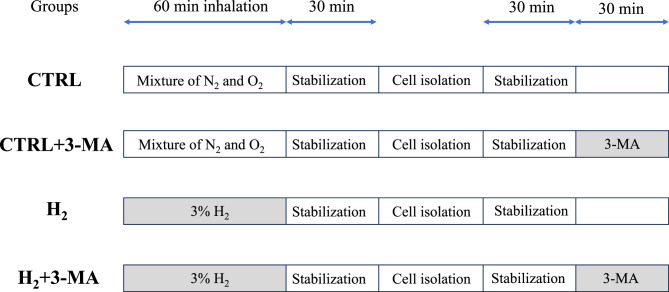
Time course of cell preparation and experimental allocation. CTRL: control; N_2_: nitrogen; O_2_: oxygen; MA: methyladenine; H_2_: hydrogen

The researchers were blinded to the allocation of cells and prepared solutions. To assess the relationship between H_2_ gas and autophagy in cardiac preconditioning, isolated cardiomyocytes were divided into four experimental groups (control [CTRL], CTRL + 3-methyladenine [MA] H_2_, and H_2_ + 3-MA groups). After stabilization, cells were incubated in a modified Tyrode’s solution with or without 10 μM of 3-MA (Sigma-Aldrich, Inc.), a selective autophagy inhibitor, for 30 min at 37 °C, and then proceeded to each experimental protocol. Figure 1 shows the graphic image of cell preparation and experimental allocation.

### Fluorescence imaging for intracellular Ca^2+^ concentrations

To investigate increase in the intracellular Ca^2+^ concentration in response to oxidative stress, a major cause of I/R injury [12], fluorescence imaging with Fluo-4 acetoxymethyl ester (Dojindo Laboratories, Kumamoto, Japan), a specific calcium indicator, was conducted using a confocal laser microscope (LSM700; Carl Zeiss, Jena, Germany). Cardiomyocytes were incubated with 5 μM Fluo-4 acetoxymethyl ester for 30 min and then perfused with 2 ml·min^−1^ of the normal Tyrode’s solution containing 100 μM of hydrogen peroxide (H_2_O_2_; [FUJIFILM Wako Pure Chemical Co.]) in the recording chamber at 23 °C. This concentration of H_2_O_2_ was shown to feasibly produce calcium overload in cardiomyocytes [13]. Viable rod-shaped cells were selected through a 40× objective lens and intermittently illuminated at a wavelength of 488 nm for 10 min. Time-course recordings were obtained from emissions above 515 nm to estimate the increasing intensity of Fluo-4 fluorescence throughout the illumination.

To investigate H_2_-induced modification of calcium kinetics, Ca^2+^ mobilization from the endoplasmic reticulum (ER) was estimated using fluorescence imaging [14]. Fluo-4-loaded cardiomyocytes were perfused with the Ca^2+^-free Tyrode’s solution for 5 min at 23 °C, followed by the solution containing 10 mM caffeine (Sigma-Aldrich, Inc.) for 1 min. ER-stored Ca^2+^ is released through ryanodine receptors and increases the intracellular Ca^2+^ transient. Time-course recordings were obtained throughout the perfusion, with the peak value estimated as the ER-stored Ca^2+^ content. Furthermore, the decay of the Ca^2+^ transient was estimated using a time constant (τ) fitted to a single exponential, considered as an excretion function of intracellular Ca^2+^ through the Na^+^-Ca^2+^ exchange system [15].

### Determination of MPTP opening

To investigate H_2_-induced modification of MPTP opening, fluorescence imaging was performed using tetramethylrhodamine ethyl ester (TMRE; [FUJIFILM Wako Pure Chemical Co.]) [11]. After incubation with 2 μM TMRE for 15 min, cells were perfused with the normal Tyrode’s solution and intermittently illuminated at a wavelength of 543 nm for 10 min at 23 °C. Time-course recordings were obtained from emissions above 570 nm through a 40× objective lens to estimate the time of MPTP opening. The opening was determined by a sharp increase in TMRE fluorescence, which is effluent from the mitochondria and is dequenched [12].

### Flow cytometry assays for signaling molecules and autophagy

To determine the upstream pathways relevant to H_2_-induced preconditioning effects, we examined the expressions of ERK and Akt, which are crucial kinases for promoting survival cascades [6, 7]. After exposure to the Ca^2+^-free or normal (1.8 mM Ca^2+^) Tyrode’s solution for 30 min, the cells were fixed and incubated with an Alexa Flour 488^®^-labeled specific antibody for ERK1/2 or Akt (Phosphoflow; Becton Dickinson Biosciences, Bedford, MA, USA) according to the manufacturer’s instructions. These antibodies are known to bind exclusively to phosphorylated residues, which are active forms of these molecules. The loaded cells were excited at a wavelength of 488 nm, filtered at 511 nm using flow cytometry (FACS II; Becton Dickinson, Bedford, MA, USA), and sorted on the basis of the forward and side scatter [16]. The expression of ERK or Akt was determined from the mean intensity of each fluorescence signal under a cellular distribution.

The emission from a low concentration of TMRE is well correlated with mitochondrial membrane potentials (ψm) [16]. After exposure to the Ca^2+^-free or normal Tyrode’s solution, cardiomyocytes were incubated in the solution containing 100 nM TMRE and were then excited at a wavelength of 488 nm and were filtered at 511 nm using flow cytometry. ψm was estimated using the mean intensity of TMRE fluorescence under a cellular distribution.

To clarify H_2_-induced activation of autophagy, we examined the expressions of autophagosomes and microtubule-associated light chain (LC) 3-II, an integral factor activating autophagy-related processes [17, 18]. Cells were exposed to the Ca^2+^-free or normal Tyrode’s solution and were then incubated with 1 μM DALGreen (Dojindo Laboratories), an indicator of autophagosome formation [19] or a Fluorescein-Isothiocyanate-labeled LC3-II antibody (Funakoshi Co., Ltd., Tokyo, Japan). The expressions of DALGreen and LC3-II were determined using flow cytometry as described above.

### Statistical analysis

All analyses were conducting on the basis of per protocols and cells which did not complete the appropriate protocols were excluded from the data analysis. The sample size was determined based on previous findings in each experimental protocol to provide a power of α error of 0.05 and β error of 0.1. In the analyses of intracellular Ca^2+^ levels, a sample size of 15 was sufficient to detect a mean difference (MD) of 40% and standard deviation (SD) of 25% [13], whereas, for the opening of the MPTP, the number of 30 was sufficient to detect the difference in survival rates between 0.25 and 0.7 in the experimental period [11]. In flow cytometry analyses, the sample size of 8 was needed for the expression of signaling molecules, with an MD of 23% and SD of 10% [9]. All experimental protocols were not adopted to repetitive measurements from the view of experimental characteristics. Experimental data were transformed into rates of change from baseline values except for those of MPTP opening. After verifying normality and homoscedasticity, between-group comparisons were conducted using a one-way analysis of variance (ANOVA) and post-hoc Tukey’s test. Mixed-effects models were used to conduct between-group comparisons, with the subject number as a random effect and the increasing rate at each point and experimental groups as a fixed effect. In contrast, repeated-measures ANOVA and post-hoc Holm’s test were performed for within-group comparisons. The time to MPTP opening was assessed using Kaplan–Meier’s curves and the log-rank test. Results are appropriately expressed as means ± SD, whereas those from the log-rank test are presented as medians [95% confidence intervals]. The number of experiments and rats are expressed as n and N. All statistical analyses were performed using the R software (version 4.0.3; R Foundation, Vienna, Austria), and data with a *P*-value less than 0.01 (two-tailed) were considered to be statistically significant in consideration of multiple comparisons.

## Results

### Changes in intracellular Ca^2+^concentrations

**Fig. 2:**
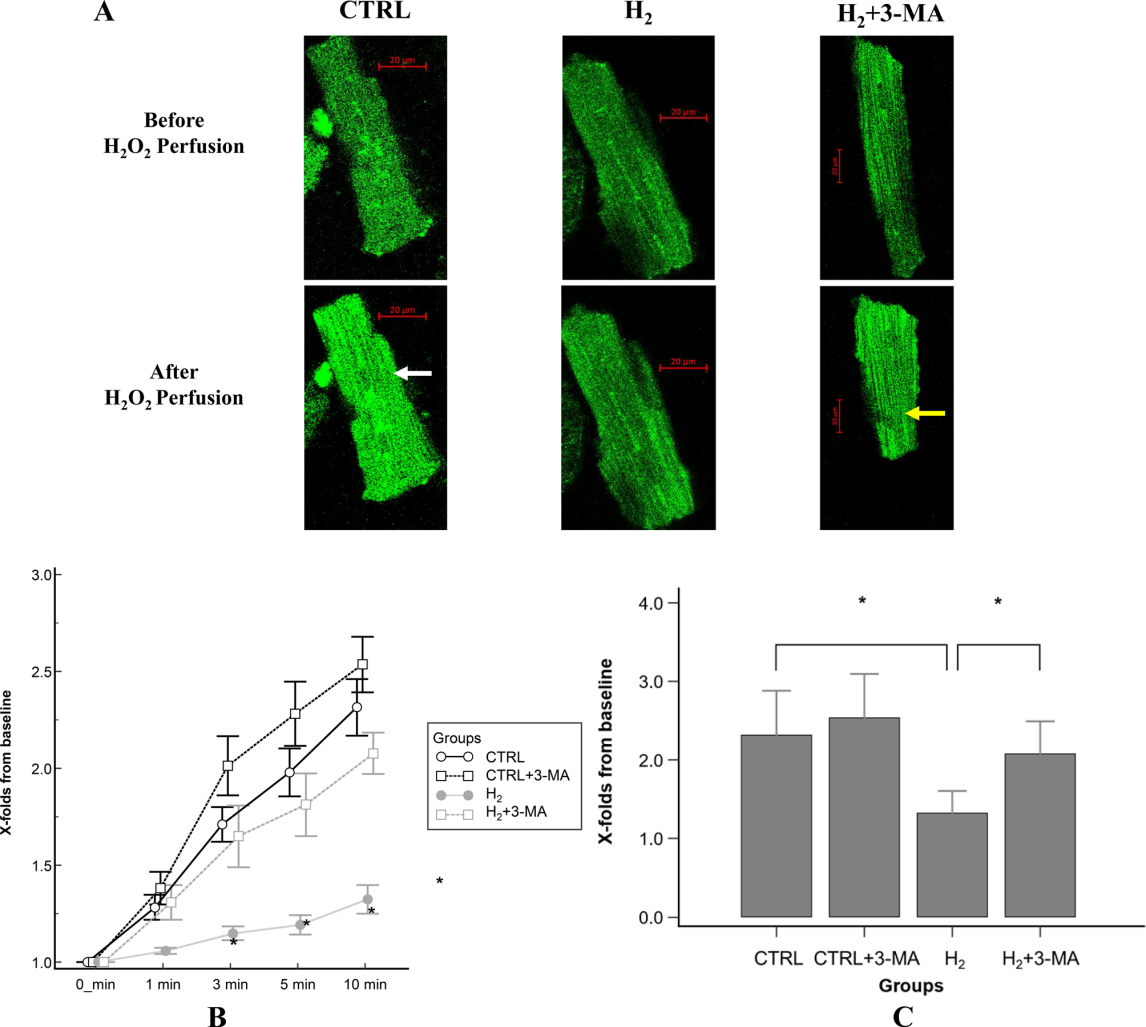
Changes in intracellular Ca^2+^ levels with 10-min H_2_O_2_ perfusion. (**A**) Representative images of fluo-4-loaded cardiomyocytes before and after 10-min H_2_O_2_ perfusion. A white arrow indicates that a cell emits intense luminescence. A yellow arrow indicates a cell produces moderate luminescence and cell-shrinking. (**B**) Time-course changes in the mean Fluo-4 fluorescence intensity during H_2_O_2_ perfusion. Error bars show standard error of the mean. The mixed-effect models revealed statistical significance between the CTRL and H_2_ groups. (**C**) Comparison of the Fluo-4 fluorescence intensity after 10-min H_2_O_2_ perfusion. Bar graphs show the increasing rate of Fluo-4 fluorescence from baseline. The rate in the H_2_ group significantly decreased relative to those in the CTRL and H_2_ + 3-MA groups. *: statistical significance with *p* < 0.001 (*n* = 15, *N* = 5 for each experimental group). CTRL: control; H_2_: hydrogen; MA: methyladenine; H_2_O_2_: hydrogen peroxide; n: the number of experiments; N: the number of rats

All experimental data were obtained from isolated cardiomyocytes. Figure 2A shows representative images of Fluo-4-loaded cardiomyocytes before and after 10-min perfusion with H_2_O_2_, indicating that intense luminescence represents an increased intracellular Ca^2+^ concentration caused by the oxidative stress. The CTRL and H_2_ + 3-MA groups increase the luminescence, whereas the H_2_ group does not change it. The within-group comparisons revealed that cells increased their fluorescence intensity in a time-dependent manner and showed statistical significance between 0 and 1 min, 1 and 2 min, and 2 and 3 min, indicating that intracellular Ca^2+^ levels were elevated in the early phase. The between-group comparisons revealed that the H_2_ group had a lower mean intensity of Fluo-4 fluorescence relative to the other groups, whereas the mixed-effect models revealed statistical significance only between the CTRL and H_2_ groups (*p* < 0.001; Fig. 2B). After 10-min perfusion with H_2_O_2_, the increasing rate of the Fluo-4 intensity from baseline reached 2.31 ± 0.57-fold in the CTRL group. H_2_ gas inhalation significantly inhibited such increase (H_2_, 1.32 ± 0.28-fold, versus [vs.] baseline; *p* < 0.001 for CTRL; Fig. 2C), which was abolished by pretreatment with 3-MA (H_2_ + 3-MA, 2.08 ± 0.42-fold, vs. baseline; *p* < 0.001 for H_2_; Fig. 2C).

**Fig. 3:**
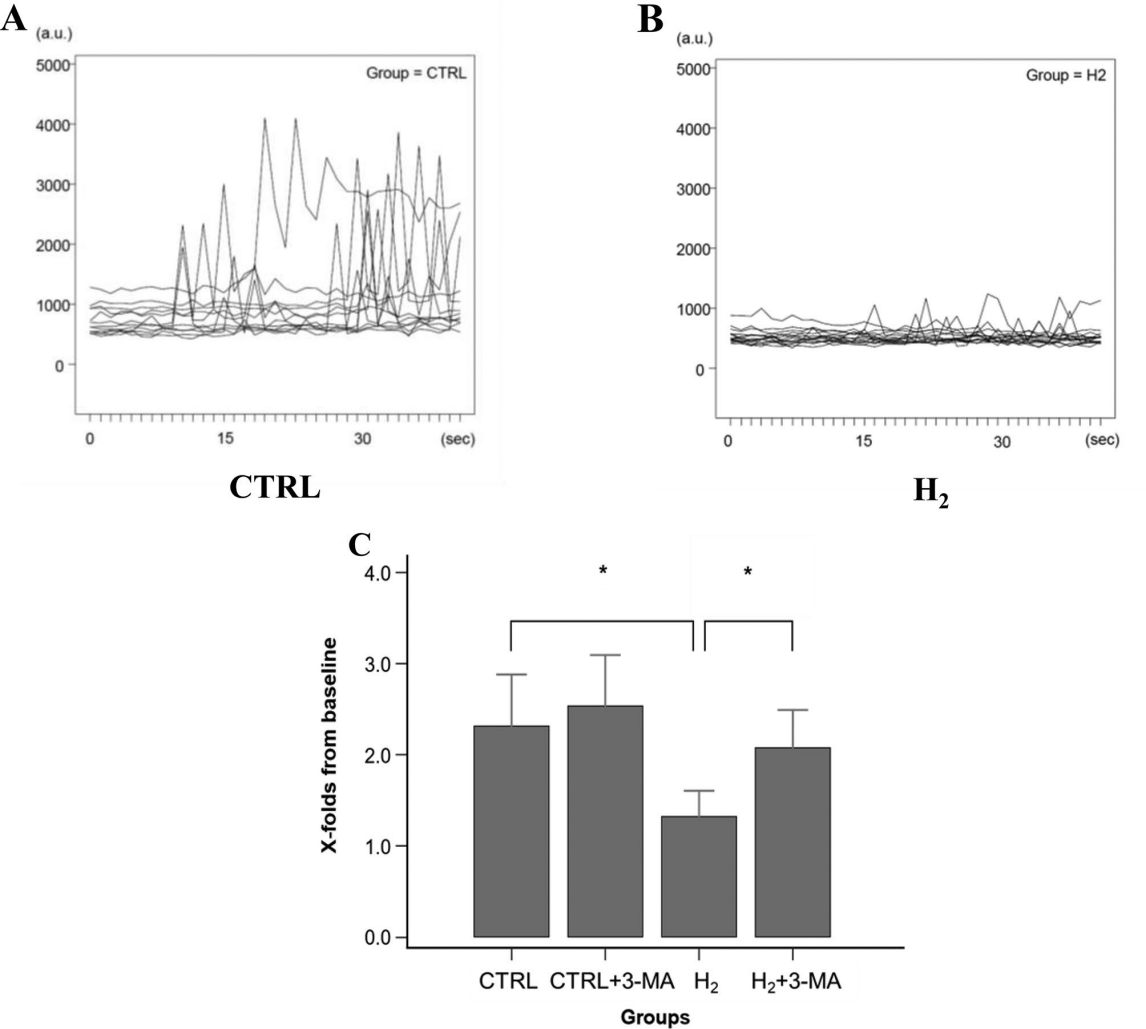
Estimation of intracellular Ca^2+^ levels after caffeine administration. (**A**,** B**) Time-course profiles of fluo-4 fluorescence after caffeine administration. (**C**) Comparison of Ca^2+^ transients from the ER. Bar graphs show the peak ratio of fluo-4 fluorescence from baseline. The ratio in the H_2_ group significantly decreased relative to those in the CTRL and H_2_ + 3-MA groups. *: statistical significance with *p* < 0.001 (*n* = 15, *N* = 5 for each experimental group). CTRL: control; H_2_: hydrogen; MA: methyladenine; ER: endoplasmic reticulum; n: the number of experiments; N: the number of rats

Figure 3A and 3B show the time-course profiles of Fluo-4 fluorescence after caffeine administration in the CTRL and H_2_ groups. Sharp increases in the fluorescence, representing Ca^2+^ transient from the ER, are observed in the CTRL group, whereas they are mostly suppressed in the H_2_ group. The comparison of Ca^2+^ transients after caffeine administration showed that the peak ratio reached 3.61 ± 0.66-fold in the CTRL group and significantly decreased in the H_2_ group (H_2_, 1.75 ± 0.27-fold, vs. baseline; *p* < 0.001 for CTRL; Fig. 3C). Pretreatment with 3-MA completely abolished the inhibitory effect induced by H_2_ gas inhalation (H_2_ + 3-MA, 3.64 ± 0.68-fold, vs. baseline; *p* < 0.001 for H_2_; Fig. 3C). Furthermore, τ computed from the exponential decay did not differ between all experimental groups, indicating that the excretion function of intracellular Ca^2+^ was unaffected by H_2_ gas inhalation (CTRL, 15.4 ± 6.6 s^−1^; CTRL + 3-MA, 12.1 ± 5.3 s^−1^; H_2_, 12.9 ± 5.3 s^−1^; H_2_ + 3-MA, 15.0 ± 8.2 s^−1^; *p* = 0.43).

### Time analysis of MPTP opening

**Fig. 4:**
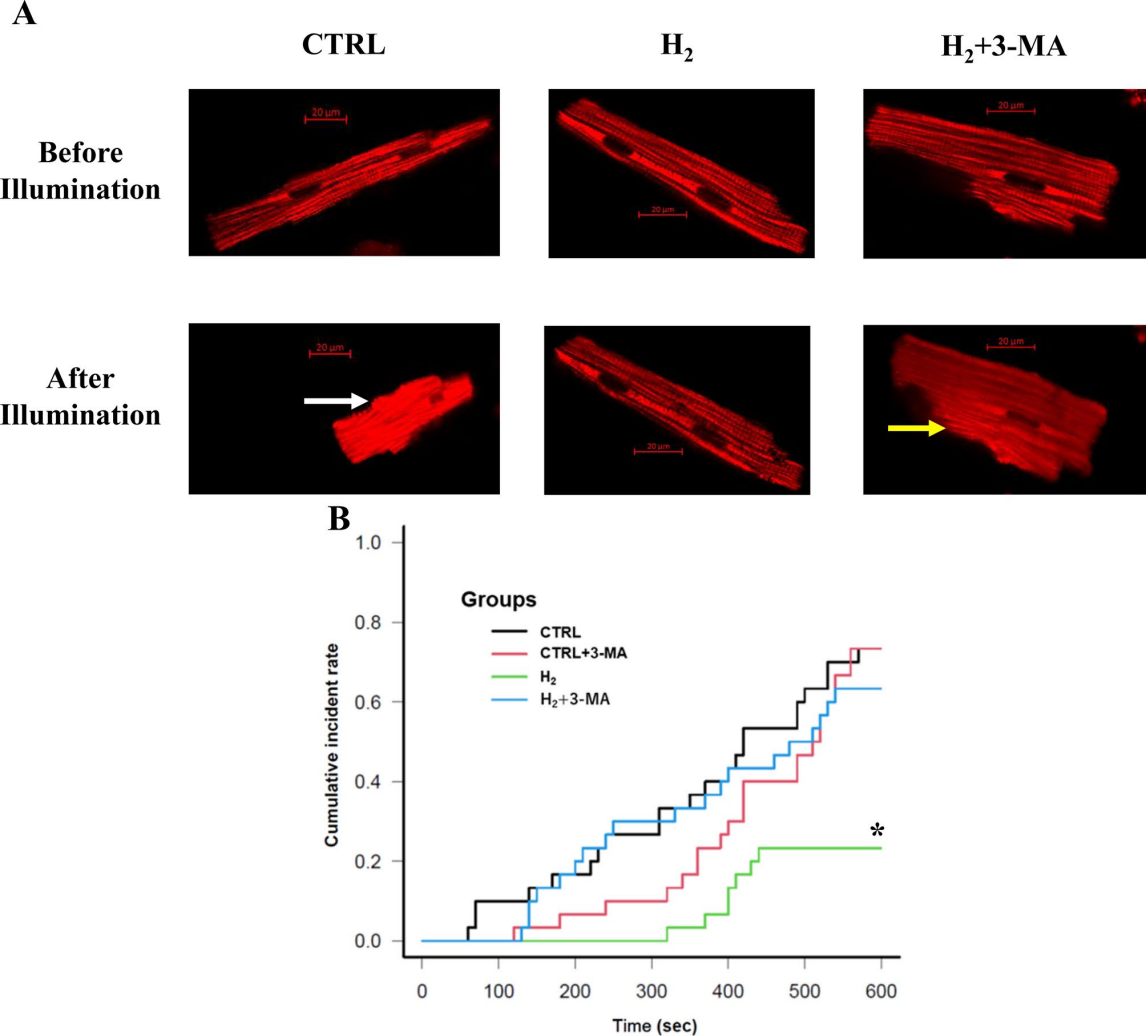
Time analysis of TMRE fluorescence for the opening of MPTP. (**A**) Representative images of the TMRE-loaded cardiomyocytes before and after 10-min perfusion. A white arrow indicates a cell produces intense luminescence and cellular hypercontraction. A yellow allow indicates that a cell produces moderate luminescence and cell-shrinking. (**B**) Kaplan–Meier curves of the opening of MPTP. The log-rank test revealed that the H_2_ group had a significantly longer time to MPTP opening relative to the CTRL and H_2_ + 3-MA groups. *: statistical significance with *p* < 0.01 for the CTRL and H_2_ + 3-MA groups (*n* = 30, *N* = 10 for each experimental group). CTRL: control; H_2_: hydrogen; MA: methyladenine; TMRE: tetramethylrhodamine ethyl ester; MPTP: mitochondrial permeability transition pore methyladenine; n: the number of experiments; N: the number of rats

Figure 4A show representative images of the TMRE-loaded cardiomyocytes before and after 10-min perfusion with the solution containing 1.8 mM Ca^2+^. The CTRL and H_2_ + 3-MA groups show increased luminescence and cell-shrinking, representing opening of the MPTP, whereas the H_2_ group does not change them. The cumulative incidences of MPTP opening were 73.3% in the CTRL and CTRL + 3-MA groups, 23.3% in the CTRL group, and 63.3% in the H_2_ + 3-MA group. The log-rank test showed that the time to MPTP opening differed significantly between the CTRL and H_2_ groups (420 s [310–530 s] vs. not applicable; *p* = 0.001; Fig. 4B). Pretreatment with 3-MA significantly shortened the opening time relative to that of the H_2_ group (H_2_ + 3-MA, 495 s [330–600 s]; *p* = 0.005 for H_2_: Fig. 4B).

### Preserved signaling molecules and ψm levels

**Fig. 5:**
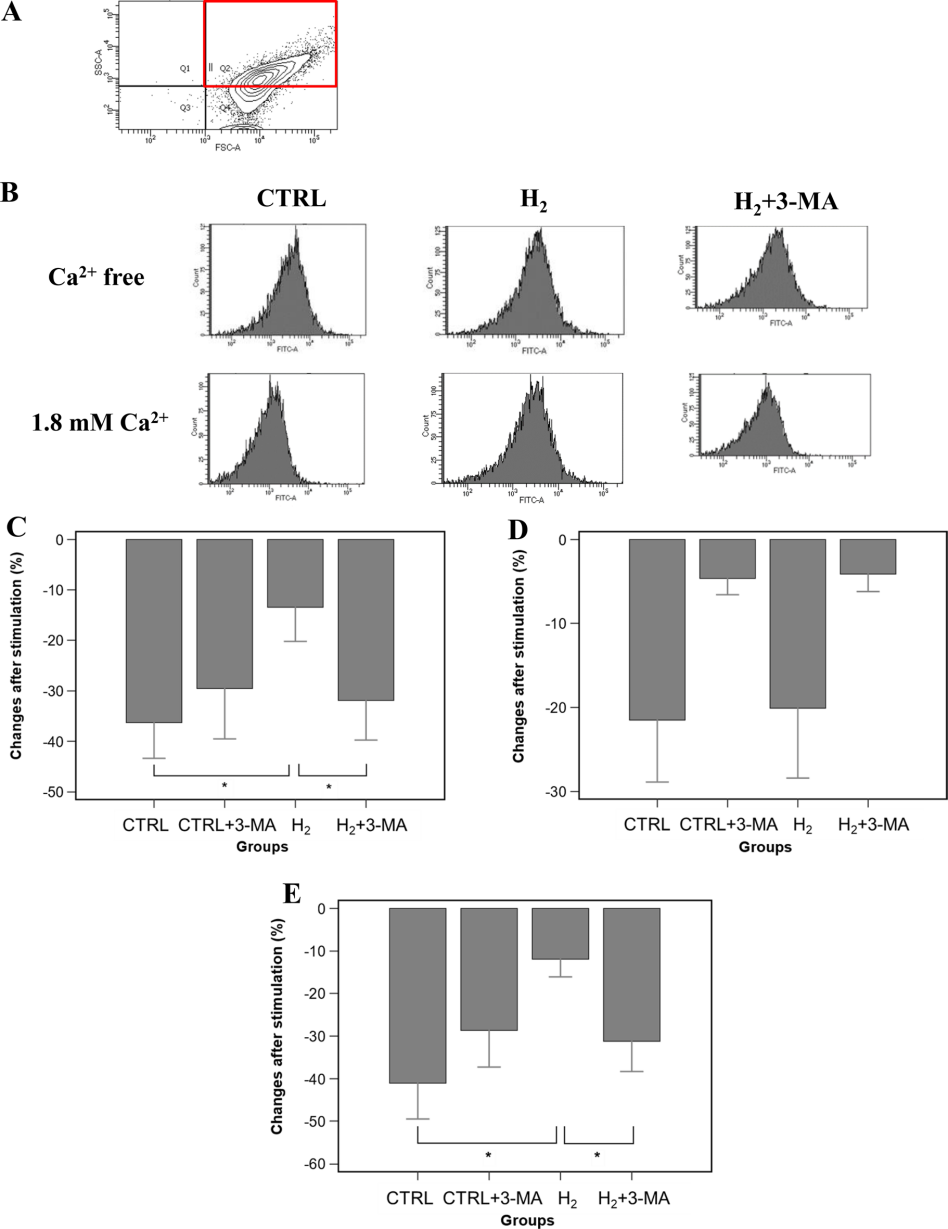
Determination of activated signaling molecules and preserved mitochondrial electrical potentials. (**A**) A representative image of cell sorting using flow cytometry. A red square shows the studied filed. (**B**) Representative profiles of cellular distribution based on ERK expression with or without 1.8 mM Ca^2+^ stimulation. (**C**) Changes in ERK expression after Ca^2+^ stimulation. Bar graphs represent percentage changes in the mean values of ERK expression. The H_2_ group preserved ERK expression, whereas the CTRL and H_2_ + 3-MA groups showed remarkably reduced ERK expression. *: statistical significance with *p* < 0.001 (*n* = 8, N = 4 for each experimental group) (**D**) Changes in AKT expression after Ca^2+^ stimulation. Bar graphs represent percent changes in the mean values of AKT expression. The H_2_ group showed remarkably reduced AKT expression, which was similar to the CTRL group. The addition of 3-MA did not affect AKT expression irrespective of H_2_ inhalation. (**E**) Changes in ψm levels after Ca^2+^ stimulation. Bar graphs represent percent changes in the mean values of ψm. The H_2_ group relatively preserved the level of ψm, whereas the CTRL and H_2_ + 3-MA groups remarkably decreased the ψm levels*: statistical significance with *p* < 0.001 (*n* = 8, *N* = 4 for each experimental group). CTRL: control; H_2_: hydrogen; MA: methyladenine; ERK: extracellular signal-regulated kinase; ψm: mitochondrial membrane potentials; n: the number of experiments; N: the number of rats

Figure 5A shows a representative image of cell sorting using flow cytometry. The right upper quarter corresponds to the studied field, representing cellular features with larger sizes and more striations. Figure 5B shows the representative profiles of the cellular distribution based on ERK expression. Stimulation with 1.8 mM Ca^2+^ moves the distribution curve to the left in the CTRL and H_2_ + 3-MA groups, indicating decreased ERK expression. In contrast, the H_2_ group does not produce such shift. Estimation was conducted using the difference between the mean values of the fluorescence intensity based on the distribution curve. Changes in ERK expression were remarkably decreased in the CTRL group after Ca^2+^ stimulation but not in the H_2_ group (CTRL, −36.3 ± 9.9%; H_2_, −13.4 ± 6.8%; vs. Ca^2+^-free exposure; *p* < 0.001; Fig. 5C). The addition of 3-MA significantly decreased ERK expression relative to that of the H_2_ group, indicating that the signaling molecule induced by H_2_ gas was mediated through autophagy (H_2_ + 3-MA, −32.0 ± 7.8%; vs. Ca^2+^-free exposure; *p* < 0.001 for H_2_; Fig. 5C). Moreover, the expression levels of Akt were concurrently reduced by Ca^2+^ stimulation in the CTRL and H_2_ groups (CTRL, −21.5 ± 7.4%; H_2_, −20.1 ± 8.3%; vs. Ca^2+^-free exposure; *p* = 0.97; Fig. 5D). The addition of 3-MA did not affect Akt expression with or without H_2_ gas inhalation (CTRL + 3-MA, −4.6 ± 2.0%; H_2_ + 3-MA, −4.1 ± 2.1%; *p* = 0.93; Fig. 5D).

Regarding changes in the levels of ψm, Ca^2+^ stimulation remarkably decreased that of the CTRL group, whereas it did not affect that of the H_2_ group (CTRL, −41.1 ± 8.5%; H_2_, −11.9 ± 4.2% vs. Ca^2+^-free exposure; *p* < 0.001; Fig. 5E). The addition of 3-MA significantly decreased the ψm level relative to that of the H_2_ group (H_2_ + 3-MA, −31.3 ± 7.1%; vs. Ca^2+^-free exposure; *p* < 0.001 for H_2_: Fig. 5E).

### Increased autophagosomes and LC3-II expression

**Fig. 6:**
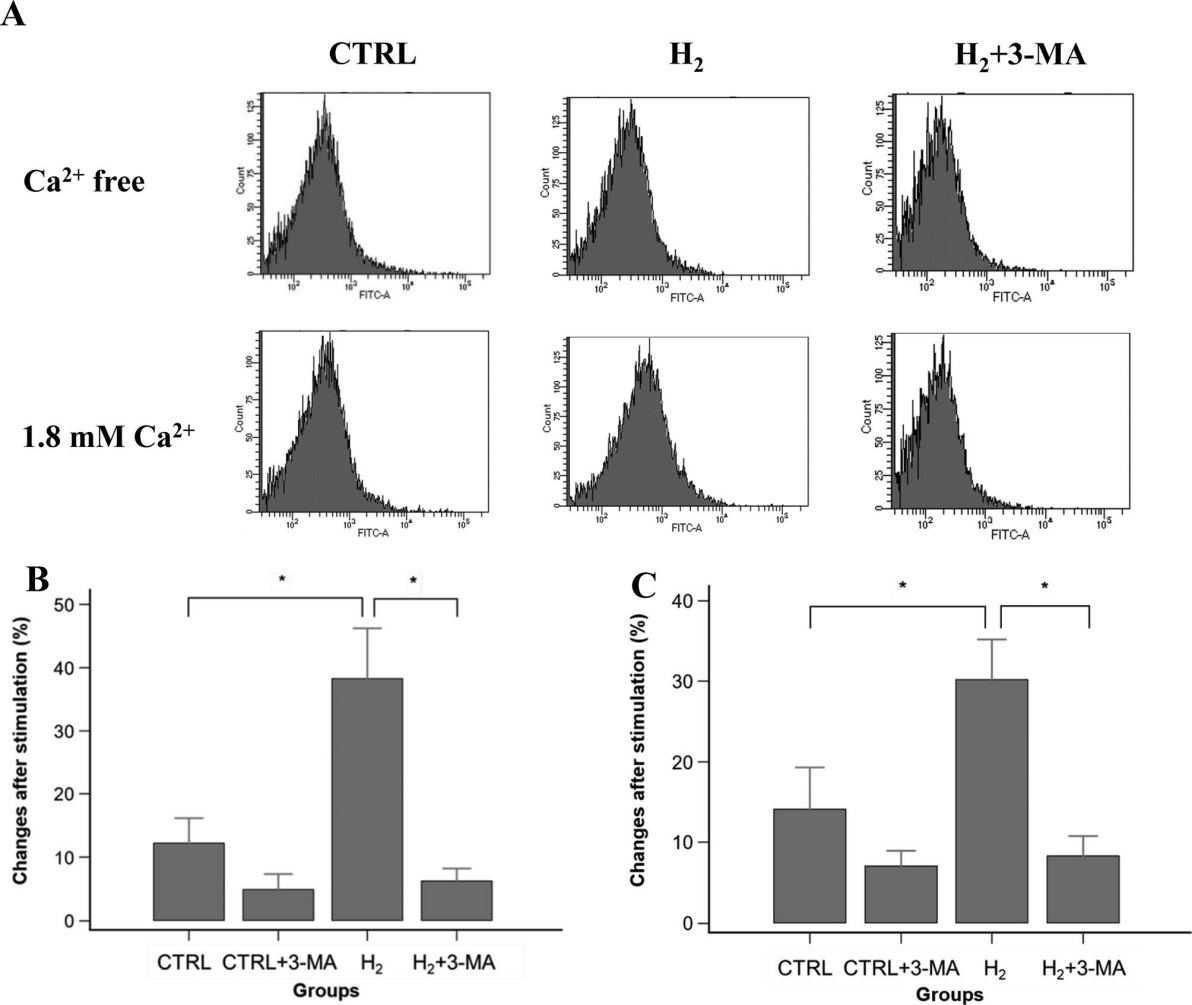
Determination of autophagy and autophagosome activation. (**A**) Representative profiles of cellular distribution based on DALGreen expression. (**B**) Changes in autophagosome formation after Ca^2+^ stimulation. Bar graphs represent percent changes in the mean values of DALGreen. DALGreen expression was significantly increased in the H_2_ group relative to the CTRL and H_2_ + 3-MA groups. *: statistical significance with *p* < 0.001 (*n* = 8, *N* = 4 for each experimental group) (**C**) Changes in LC3-II expression after Ca^2+^ stimulation. Bar graphs represent percent changes in the mean values of LC3-II expression. LC3-II expression was significantly increased in the H_2_ group relative to the CTRL and H_2_ + 3-MA groups. *: statistical significance with *p* < 0.001. (*n* = 8, *N* = 4 for each experimental group). CTRL: control; H_2_: hydrogen; MA: methyladenine; LC: light chain; n: the number of experiments; N: the number of rats

Figure 6A shows representative profiles of cellular distribution based on DALGreen expression using flow cytometry. Stimulation with 1.8 mM Ca^2+^ moves the distribution curve to the right in the H_2_ group, indicating increased DALGreen expression. Estimation was conducted using the difference between the mean values of the fluorescence intensity based on the distribution curve. Changes in DALGreen were increased in the CTRL and H_2_ groups after Ca^2+^ stimulation and further increase in the fluorescence was shown in the H_2_ group (CTRL, 12.2 ± 4.0%; H_2_, 38.3 ± 7.9%, vs. Ca^2+^-free exposure; *p* < 0.001; Fig. 6B). This indicated that autophagosome formation was initiated by Ca^2+^ stimulation and further accelerated by H_2_ gas inhalation. The addition of 3-MA suppressed autophagosome formation in cardiomyocytes with or without H_2_ gas inhalation, although a statistically significant difference between the H_2_ and H_2_ + 3-MA groups was noted (CTRL + 3-MA, 4.9 ± 2.5%; H_2_ + 3 MA, 6.2 ± 2.0%, vs. Ca^2+^-free exposure; *p* = 0.02 for CTRL or *p* < 0.001 for H_2_; Fig. 6B). Furthermore, Ca^2+^ stimulation increased the expression levels of LC3-II in the CTRL and H_2_ groups and exhibited further increased expression in the H_2_ group (CTRL, 14.3 ± 5.2%; H_2_, 30.3 ± 4.9%, vs. Ca^2+^-free exposure; *p* < 0.001; Fig. 6C). The addition of 3-MA significantly decreased LC3-II expression with or without H_2_ gas inhalation (CTRL + 3-MA, 7.1 ± 1.9%; H_2_ + 3-MA, 8.3 ± 2.4%, vs. Ca^2+^-free exposure; *p* = 0.006 for CTRL or *p* < 0.001 for H_2_; Fig. 6C).

## Discussion

The current study demonstrated that preconditioning with 3% H_2_ gas for 60 min effectively inhibited the H_2_O_2_-perfused calcium overload and Ca^2+^ mobilization from the ER and prolonged to the time to MPTP opening. Furthermore, H_2_ gas inhalation preserved the expression of upstream signaling molecules of ERK and LC3-II and the electrical potentials in the mitochondria. These protective effects were completely abolished by pretreatment with the autophagy inhibitor 3-MA. These findings are congruent with our hypothesis that H_2_ gas inhalation has a cardiac preconditioning effect with the combined activation of cellular signaling cascades and autophagy and are novel in that H_2_ gas possesses a cellular mechanism for alleviating oxidative stress other than scavenging hydroxyl radicals.

Following the demonstration that H_2_ gas was a selective radical scavenger for hydroxyl radicals and alleviated oxidative stress during I/R injury [2], several clinical trials have shown that H_2_ gas inhalation is a successful treatment for I/R injury-induced cardiac damage [3, 4]. As previous studies mainly focused on concurrent or subsequent administration of H_2_ gas for ischemic events, preventive intervention with H_2_ gas inhalation–a more practical approach–remains unconfirmed owing to limited knowledge. This study demonstrated that prior inhalation of 3% H_2_ gas had a preconditioning effect against oxidative stress-induced myocardial damage and was mainly attributed to suppressing the increase in intracellular Ca^2+^ levels and Ca^2+^ mobilization from the ER. This action mechanism differs from scavenging hydroxyl radicals and leads to preservation of ψm and resistance to MPTP opening, which is strictly regulated by intracellular Ca^2+^ levels and the upstream signaling molecules [6, 7]. Taken together, the root mechanism of the preconditioning effect induced by H_2_ gas is to preserve mitochondrial homeostasis through the maintenance of intracellular Ca^2+^ levels and activate cellular survival pathways, including ERK. These findings were consistent with the previous report that some physiologically-active gas exerted a cardio-protective effect through the activation of ERK [20].

Several studies have mentioned the relationship between autophagy and cardio-protection, with which several signaling pathways are involved. The mitogen-activated protein kinase (MAPK)/ERK pathway promotes autophagy to remove waste products and maintain intracellular homeostasis [9]. In contrast, the phosphatidylinositol-3 kinase (PI3K)/Akt pathway inhibits autophagy to avoid excessive degradation of organelles [10, 21]. Despite their discrepant involvement with the autophagy process, both signaling pathways are key factors in cardiac preconditioning [6, 7]. On the basis of the significant increase in autophagosome formation and LC3-II expression, H_2_ gas inhalation predominantly induced autophagy with ERK activation in this study. However, positive Akt expression, a counterpart of ERK activation, was not induced by H_2_ gas inhalation. This might be attributable to our experimental feature of cardiac preconditioning, because H_2_ gas inhalation was completed before myocardial damage occurred. In fact, H_2_ gas inhalation can activate the PI3K/Akt pathway to inhibit autophagy, provided that cellular damage preceded the inhalation [22].

The current study demonstrates the preconditioning effect of H_2_ gas inhalation mediated through the combined effects of the MAPK/ERK pathway and autophagy. Besides ERK, autophagy can regulate MPTP opening by preserving mitochondrial homeostasis associated with potassium channels [23]. ERK is considered as an upstream factor in the development of autophagy [6]; however, we demonstrated the mutual interaction between ERK and autophagy because an autophagy inhibitor completely suppressed H_2_-induced ERK expression. Thus, H_2_ gas inhalation concurrently activates both systems and profoundly preserves cellular homeostasis, thereby producing a robust preconditioning effect in the myocardium. Recent studies suggesting the interaction between the MAPK/ERK pathway and autophagy support our findings and indicate a potential treatment target for several pathological conditions [10, 24].

The current study has some limitations. First, a 30-min stabilization duration after H_2_ gas inhalation was determined for the memory phase of cardiac preconditioning that is needed for the development of cellular signaling pathways [25]. The residual effect as a radical scavenger may have been related to the inhalation schedule because the myocardial concentrations of H_2_ were not measured. However, as H_2_ gas reportedly disappears from the myocardium minutes after discontinuing inhalation, it could not serve as a radical scavenger in this study [26]. Second, lower concentrations of H_2_ gas may have produced similar results but were not investigated in this study. A concentration of 3% was determined for safety margin and carrier gas use, although the dosage is generally restricted to 4% for inhalational use [27]. We primarily focused on exploratory research to assess the effectiveness of inhaled H_2_ gas at a relatively high concentration; therefore, further research on the dose-response effects of inhaled H_2_ gas on cardiac preconditioning is warranted. Third, the stored content of Ca^2+^ in the ER is known as the mainstream of calcium kinetics at I/R injury, heavily released into the cytosol at reperfusion [28, 29]. However, the influx of extracellular Ca^2+^ through the reverse-mode Na^+^-Ca^2+^ exchange (NCX) or transient receptor potential canonical channels might have been involved in the increased intracellular Ca^2+^ concentration in this study [13]. Furthermore, the conditions of the reverse-mode NCX might influence our findings that H_2_ gas inhalation did not change the excretion of intracellular Ca^2+^ through the forward-mode NCX. Fourth, we assessed the expression of ERK and Akt as representative molecules that generate cellular survival cascades in cardiomyocytes. The Janus kinase/signal transducer and activator of transcription pathway reportedly possesses a late-phase preconditioning effect after cytokine stimulation [30]. This finding contradicts our premise that the early preconditioning effect occurs within several hours after H_2_ gas inhalation. Fifth, western blot analysis is a gold standard of estimating signaling molecules, but it includes outcomes from dead cells induced by stimulation and may underestimate those form our experimental protocols using isolated cells [31, 32]. We therefore conducted a sequence of flow cytometry analyses and obtained results from the sorted cardiomyocytes with living potential.

## Conclusions

The preconditioning with inhaled H_2_ gas confers a cardio-protective effect against oxidative stress, mediated through the activation of the MAPK/ERK pathway, but not the PI3K/Akt, pathway. Autophagy activation, which helps maintain cellular or mitochondrial homeostasis, facilitates the expression of the H_2_-induced survival cascades. Preventive intervention with H_2_ gas is valuable for ensuring feasibility and safety and has clinical significance in perioperative medicine, especially in patients undergoing cardiac surgery.

## Data Availability

The datasets used and/or analyzed during the current study are available from the corresponding author on reasonable request.

## Abbreviations

I/R: Ischemia/Reperfusion
ROS: Reactive oxygen species
H_2_: Hydrogen
ERK: Extracellular signal-regulated kinase
MPTP: Mitochondrial permeability transition pore
H_2_O_2_: Hydrogen peroxide
ER: Endoplasmic reticulum
τ: Time constant
TMRE: Tetramethylrhodamine ethyl ester
ψm: Mitochondrial membrane potentials
LC: Microtubule-associated light chain
